# Supplementary material for: Aptameric hirudins as selective and reversible EXosite-ACTive site (EXACT) inhibitors
Source: Nat Commun. 2024 May 10;15:3977. doi: 10.1038/s41467-024-48211-6 (PMC11087511; doi:10.1038/s41467-024-48211-6)
Supplement: Supplementary file 1 — Supplementary Information [file 41467_2024_48211_MOESM1_ESM.pdf]

Supplementary Information for

Aptameric hirudins as selective and reversible EXosite-ACTive site (EXACT) inhibitors

Haixiang Yu, Shekhar Kumar, James W Frederiksen, Vladimir N Kolyadko, George Pitoc, Juliana Layzer, Amy Yan, Rachel Rempel, Samuel Francis, Sriram Krishnaswamy* & Bruce A Sullenger*

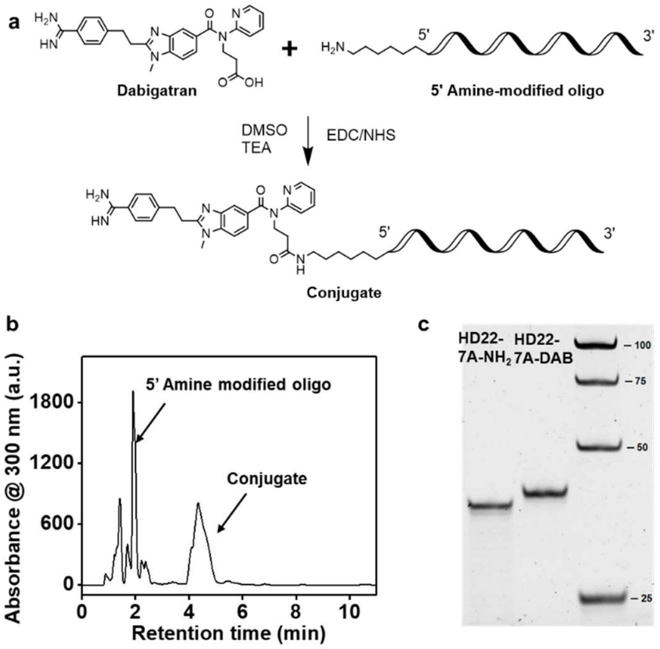

Supplementary Figure 1: Synthesis and purification of the HD22-DAB conjugates. **a,** The HD22-DAB conjugates were synthesized from dabigatran and 5' Amine modified HD22 derivatives via EDC/NHS conjugation following by (**b**) HPLC purification where the conjugated fraction from 4-5min was collected. **c,** The purity of the final products was validated by 15% denaturing PAGE.

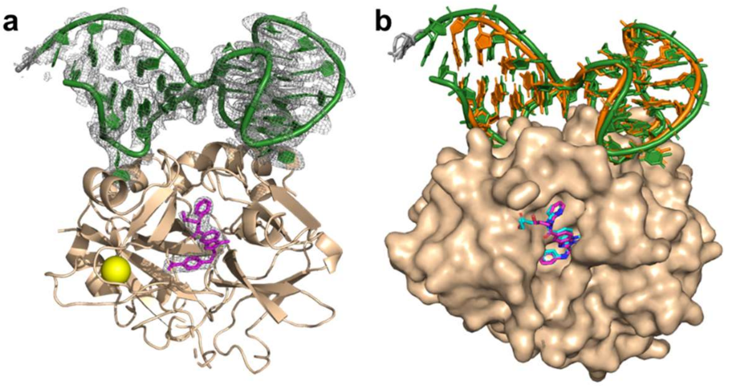

Supplementary Figure 2: Crystal structure of HD22-7A-DAB (HD22 aptamer-green, partial of the linker-grey and DAB-purple) binding to thrombin S195A (wheat). **a,** Composite omit (2Fo − Fc electron-density map, gray mesh, contoured at 1.0σ) map showing the electron density of HD22-7A-DAB. Thrombin is shown in the standard orientation. The sodium ion is shown as a yellow sphere. **b,** the structure superpositioned with previously published HD22 (orange, PDBID 4I7Y) and dabigatran (cyan, PDBID 1KTS) structures binding to thrombin.

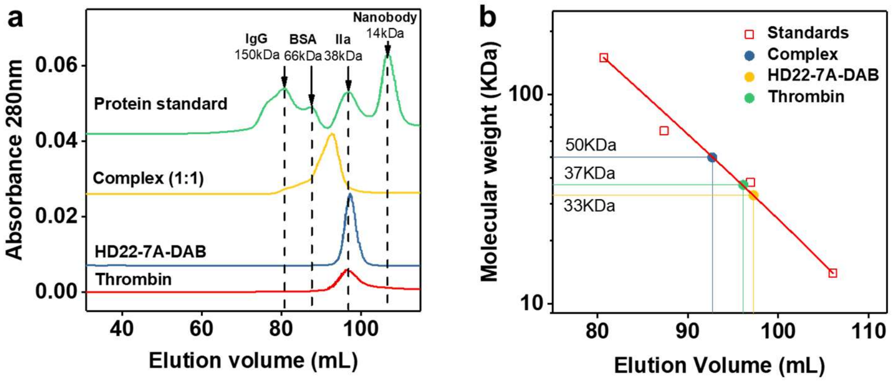

Supplementary Figure 3: Determination of binding stoichiometry of HD22-7A-DAB to thrombin using size exclusion chromatography. **a**, the elusion profiles of protein standard, HD22-7A-DAB (1.25 nmole), thrombin (1.25nmole) and HD22-7A-DAB thrombin mixture (yellow: 1.25 nmole each). **b**, apparent molecular weight of HD22-7A-DAB, thrombin and HD22-7A-DAB thrombin complex. When HD22-7A-DAB complexes with thrombin, the total surface area exposed to the solvent is reduced. Thus it appears to behave as a smaller molecule in the column, which leads to an apparent molecular weight (50KDa) that's lower than the sum of its components (70KDa), indicating a 1:1 binding stoichiometry.

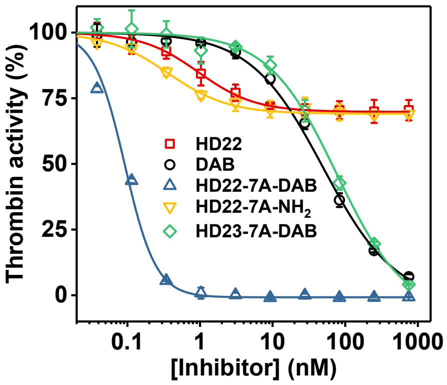

Supplementary Figure 4: Synergy of HD22-7A-DAB. The thrombin inhibition activity of HD22-7A-DAB was compared to HD23-7A-DAB, and HD22-7A-NH_2-mediated inhibition where the aptamer or the small molecule inhibitor was replaced with an inactive moiety, respectively. HD22 and DAB were used as controls in the assay. Data are presented as mean values +/- SD calculated from three independent experiments (n=3).

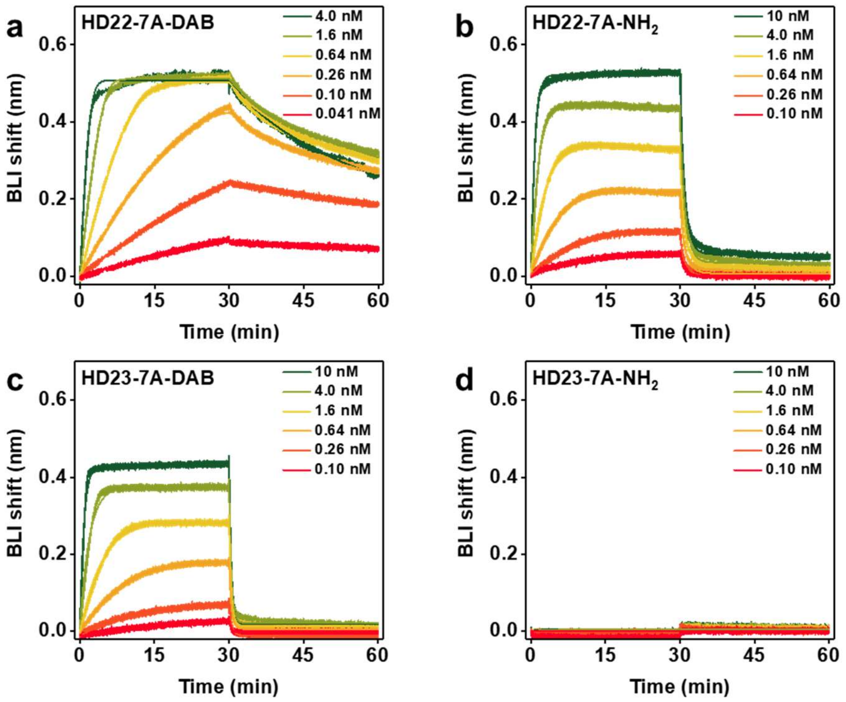

e

	k_{on} ($M^{-1}s^{-1}$)	k_{off} (s^{-1})	K_D (M)
**HD22**-7A-**DAB**	3.9×10^{7}	2.1×10^{-3}	5.4×10^{-11}
**HD22**-7A-NH_2	2.6×10^{6}	1.7×10^{-2}	6.5×10^{-9}
HD23-7A-**DAB**	2.0×10^{6}	3.8×10^{-2}	1.9×10^{-8}
HD23-7A-NH_2	ND	ND	ND

Supplementary Figure 5: Determination of binding kinetics and dissociation constants by bio-layer interferometry. **a-d**, Interactions of immobilized HD22-7A-DAB (a), HD22-7A-NH_2 (b), HD-23-7A-DAB (c), and HD23-7A-NH_2 (d) with different concentrations of thrombin. **e**, the fitted binding kinetics and dissociation constants of HD22-7A-DAB and derivatives.

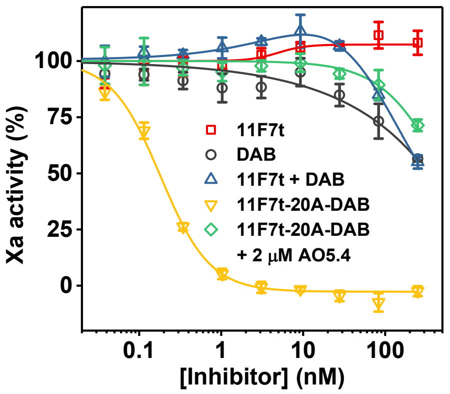

Supplementary Figure 6: Factor Xa inhibition by 11F7t-20A-DAB and other inhibitors in fluorescent peptidyl substrate cleavage assay. 11F7t-20A-DAB (yellow) showed a significantly higher inhibitory effect of factor Xa than DAB, 11F7t, or their equimolar mixture, and 11F7t-20A-DAB can be efficiently reversed by addition of an antidote oligonucleotide (AO5.4) (green). Data are presented as mean values +/- SD calculated from three independent experiments (n=3).

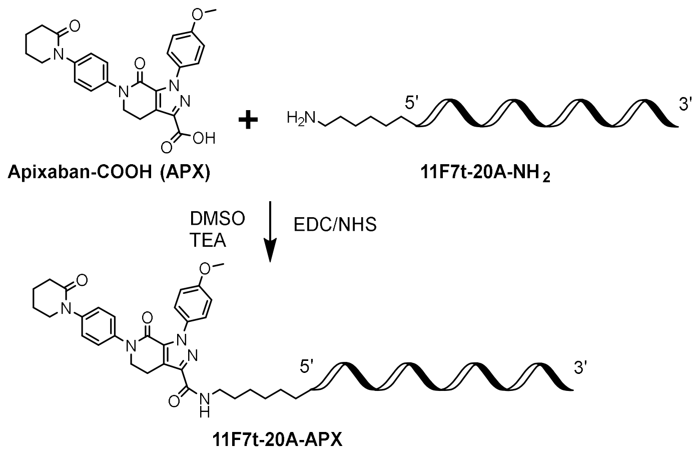

Supplementary Figure 7: Synthesis of the 11F7t-20A-APX via EDC/NHS conjugation.

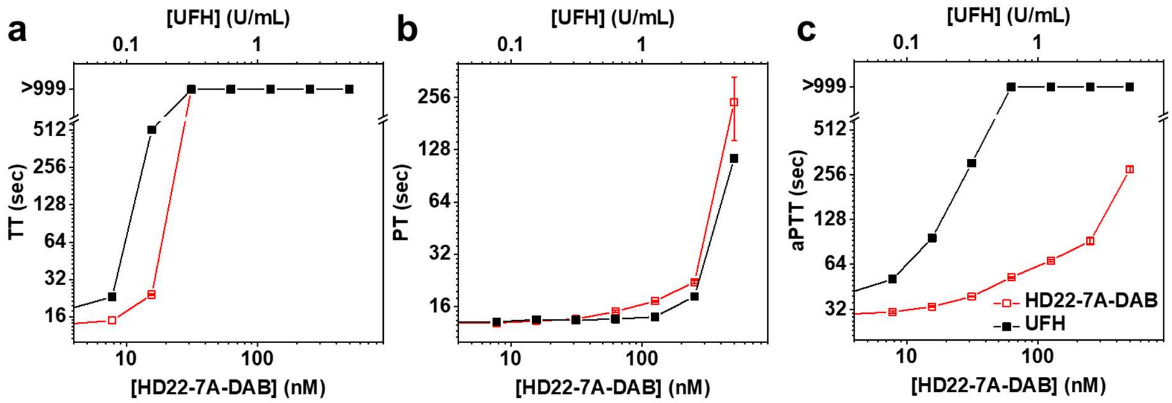

Supplementary Figure 8: Anticoagulation activity of EXACT inhibitor HD22-7A-DAB (red) in comparison with UFH (black). Different concentrations HD22-7A-DAB or UFH were added to normal human plasma and incubated for 5 min at 37˚C and **a**, Thrombin time (TT), **b**, Prothrombin time (PT), and **c**, Activated partial thromboplastin time (aPTT) assays were performed to characterize anticoagulant efficacy. To characterize antidote reversal of HD22-7A-DAB, another 5-min incubation of antidote oligo (2 μM) with the plasma-HD22-7A-DAB mixture was performed. In the TT assay, plasma did not clot within the measurable range of the coagulation analyzer (999 sec) when the concentration of HD22-7A-DAB exceeds 16 nM. Data are presented as mean values +/- SD calculated from two independent experiments (n=2).

Supplementary Table 1. Oligonucleotides used in this work.

Oligo ID	Sequence (5’ → 3’)
**HD22**	AGTCCGTGGTAGGGCAGGTTGGGGTGACT
**HD22-0A-DAB**	DAB-C_6H_{12}-AGTCCGTGGTAGGGCAGGTTGGGGTGACT
**HD22-2A-DAB**	DAB-C_6H_{12}-A(2)AGTCCGTGGTAGGGCAGGTTGGGGTGACT
**HD22-5A-DAB**	DAB-C_6H_{12}-A(5)AGTCCGTGGTAGGGCAGGTTGGGGTGACT
**HD22-7A-DAB**	DAB-C_6H_{12}-A(7)AGTCCGTGGTAGGGCAGGTTGGGGTGACT
**HD22-7A-NH_2**	NH_2-C_6H_{12}-A(7)AGTCCGTGGTAGGGCAGGTTGGGGTGACT
**HD23-7A-DAB**	DAB-C_6H_{12}-A(7)AGTCCGTAATAAAGCAGGTTAAAATGACT
**HD22-10A-DAB**	DAB-C_6H_{12}-A(10)AGTCCGTGGTAGGGCAGGTTGGGGTGACT
**HD22-15A-DAB**	DAB-C_6H_{12}-A(15)AGTCCGTGGTAGGGCAGGTTGGGGTGACT
**HD22-20A-DAB**	DAB-C_6H_{12}-A(20)AGTCCGTGGTAGGGCAGGTTGGGGTGACT
**HD22-25A-DAB**	DAB-C_6H_{12}-A(25)AGTCCGTGGTAGGGCAGGTTGGGGTGACT
**HD22-30A-DAB**	DAB-C_6H_{12}-A(30)AGTCCGTGGTAGGGCAGGTTGGGGTGACT
**Bio-HD22-7A-DAB**	DAB-C_6H_{12}-A(7)AGTCCGTGGTAGGGCAGGTTGGGGTGACT-TEG-biotin
**Bio-HD22-7A-NH_2**	NH_2-C_6H_{12}-A(7)AGTCCGTGGTAGGGCAGGTTGGGGTGACT-TEG-biotin
**Bio-HD23-7A-DAB**	DAB-C_6H_{12}-A(7)AGTCCGTAATAAAGCAGGTTAAAATGACT-TEG-biotin
**Bio-HD23-7A-NH_2**	NH_2-C_6H_{12}-A(7)AGTCCGTAATAAAGCAGGTTAAAATGACT-TEG-biotin
**AO1**	AGTCACCCCAACCTGCCCTACCACGGACTTTTTT
**AO2**	AGTCACCCCAACCTGCCCTACCACGGACTTTTTTTT
**AO3**	ACCCCAACCTGCCCTACCACGGACTTTTTTTT
**AO4**	CAACCTGCCCTACCACGGACTTTTTTTT
**AO5**	CTGCCCTACCACGGACTTTTTTTT
**AO6**	CCTACCACGGACTTTTTTTT
**AO7**	CCACGGACTTTTTTTT
**AO8**	GGACTTTTTTTT
**AO9**	TTTTTTT
**11F7t-20A-DAB**	DAB-C_6H_{12}-<br>$^{OMe}A(20)^{r}G^{r}A^{r}G^{r}A^{r}G^{F}C^{F}C^{F}C^{c}C^{r}A^{r}G^{F}C^{r}G^{r}A^{r}G^{r}A^{F}U^{r}A^{r}A^{F}U^{r}A^{F}C^{F}U^{F}U^{r}G^{r}G^{F}C^{F}C^{F}C^{F}C^{r}G^{F}C^{F}U^{F}C^{F}U^{F}U$
**11F7t-20A-APX**	APX-C_6H_{12}-<br>$^{OMe}A(20)^{r}G^{r}A^{r}G^{r}A^{r}G^{F}C^{F}C^{F}C^{c}C^{r}A^{r}G^{F}C^{r}G^{r}A^{r}G^{r}A^{F}U^{r}A^{r}A^{F}U^{r}A^{F}C^{F}U^{F}U^{r}G^{r}G^{F}C^{F}C^{F}C^{F}C^{r}G^{F}C^{F}U^{F}C^{F}U^{F}U$
**11F7t**	$^{r}G^{r}A^{r}G^{r}A^{r}G^{F}C^{F}C^{F}C^{c}C^{r}A^{r}G^{F}C^{r}G^{r}A^{r}G^{r}A^{F}U^{r}A^{r}A^{F}U^{r}A^{F}C^{F}U^{F}U^{r}G^{r}G^{F}C^{F}C^{F}C^{F}C^{r}G^{F}C^{F}U^{F}C^{F}U^{F}U$
**AO5.4**	TATTATCTCGCTGGGGCTCTCTTTTTTTTTTTTTTTTTTT

NH_2-C_6H_{12}: Amine modifier with C6 linker, DAB: dabigatran conjugation, TEG-biotin: biotin modifier with triethylene glycol spacer, ^{OMe}A: 2’ O-methyl modified A, ^{r}G: ribo-G, ^{r}A: ribo-A, ^{F}C: 2’ Fluoro modified C, ^{F}U: 2’ Fluoro modified U.

Supplementary Table 2. Data collection and refinement statistics (molecular replacement)

	HD22-7A-DAB-IIa<br>PDB ID: 8TQS
**Data collection**	
Wavelength (Å)	0.9793
Space group	P 41 21 2
Cell dimensions	
a, *b*, *c* (Å)	81.63, 81.63,190.51
α, β, γ (°)	90, 90, 90
Resolution (Å)	41.17 – 2.21 (2.2-26)
R_{merge}	0.127(1.3)
$I / \sigma I$	11.7(1.8)
Completeness (%)	99.23(91.8)
Redundancy	14.4(12.1)
**Refinement**	
Resolution (Å)	2.2
No. unique reflections	33265 (1614)
R_{work} / R_{free}	0.204/0.24
No. atoms	31011
Protein + RNA	3000
Ligand/ion	63
Water	14
B-factors	
Protein + RNA	72.2
Ligand/ion	86.5
Water	62.4
Clashscore	7.62
R.m.s. deviations	
Bond lengths (Å)	0.011
Bond angles (°)	2.03
Ramachandran	
Favored (%)	96.85
Allowed (%)	3.15
Outliers (%)	0

Supplementary Note 1. IC_{50} in fluorescent peptidyl substrate cleavage assays. In this work, IC_{50} instead of K_i is compared due to the different inhibition mechanism between exosite and active site binding inhibitors. IC_{50} of an active site inhibitor is higher than its K_i due to the competition between the inhibitor and the substrate. One the other hand, IC_{50} of an exosite inhibitor is similar to its K_i. We believe that IC_{50}, compared to K_i, better represents the potency of different inhibitors in the presence of substrate.

We also found that IC_{50}s of several EXACT inhibitors are lower than half of the presumed protease concentration (0.25 nM), which conflicts with the 1:1 binding stothiometry confirmed by size exclusion chromatography. Because a low concentration (0.5 nM) of protease is used in the assay, a portion of thrombin may be lost or become inactive due to adsorbtion to plastic wares such as pipette tips, tubes, and microplate, and the actual active thrombin concentration in the assay may be lower than 0.5 nM. From the experiment shown in Figure 2d, we use global fitting to estimate the active protease concentration to be 0.19 nM (see below). Under such estimation, an IC_{50} as low as 0.1 nM does not conflict with the 1:1 binding stothiometry. Notably, since comparison of different inhibitors were always performed in the same experiment, the protease concentration in different samples is the same and should not affect our conclusions.

Supplementary Note 2. Two-step binding model of EXACT inhibitors. The following two-step model describes the binding equilibriums between a bivalent EXACT inhibitor with the protease. Figure 1d represents the two possible pathways for the stepwise ligation of thrombin (E) with the EXACT inhibitor (AI) containing the aptamer (A) conjugated to DAB (I) by a linker. $K_{E,A}$ and $K_{E,I}$ represent the equilibrium dissociation constants for the initial ligation of either the aptamer or DAB to thrombin. As supported by the data, these binding constants are assumed to be equivalent to those seen with the monovalent ligands. Initial binding is followed by a dimensionless unimolecular step in which either I binds to the active site when A is already bound or A binds to the exosite when I is already bound to give a common doubly ligated product (AEI). As defined, AEI is increasingly favored at smaller values $K_{EA,I}$.

The relationship between the concentrations of E, AI, AIE, EAI, and AEI can be determined by three dissociation constants, $K_{E,A}$, $K_{E,I}$, and $K_{EA,I}$ with following equilibriums:

$$AIE = \frac{E \cdot AI}{K_{E,A}};\ EAI = \frac{E \cdot AI}{K_{E,I}};\ AEI = \frac{E \cdot AI}{K_{E,A} \cdot K_{EA,I}}$$

The composition of bivalent binding complex (AEI) in all complexs (AIE, EAI, and AEI) can be determined as:

$$\frac{\frac{1}{K_{E,A} \cdot K_{EA,I}}}{\frac{1}{K_{E,A}} + \frac{1}{K_{E,I}} + \frac{1}{K_{E,A} \cdot K_{EA,I}}}$$

With a given total concentration of protease and EXACT inhibitor to be E_t and AI_t, respectively:

$$E_t = E + AIE + EAI + AEI = E \cdot AI \cdot (\frac{1}{AI} + \frac{1}{K_{E,A}} + \frac{1}{K_{E,I}} + \frac{1}{K_{E,A} \cdot K_{EA,I}})$$

and

$$AI_t = AI + AIE + EAI + AEI = AI + E_t - E = \frac{AI + [\left(\frac{1}{K_{E,A}} + \frac{1}{K_{E,I}} + \frac{1}{K_{E,A} \cdot K_{EA,I}}\right) \cdot AI] \cdot (AI + E_t)}{1 + \left(\frac{1}{K_{E,A}} + \frac{1}{K_{E,I}} + \frac{1}{K_{E,A} \cdot K_{EA,I}}\right) \cdot AI}$$

From the above equations, AI can be determined as:

$$\frac{\sqrt{[\left(\frac{1}{K_{E,A}} + \frac{1}{K_{E,I}} + \frac{1}{K_{E,A} \cdot K_{EA,I}}\right) \cdot (E_t - AI_t) + 1]^2 + 4 \cdot (\frac{1}{K_{E,A}} + \frac{1}{K_{E,I}} + \frac{1}{K_{E,A} \cdot K_{EA,I}}) \cdot AI_t} - [(\frac{1}{K_{E,A}} + \frac{1}{K_{E,I}} + \frac{1}{K_{E,A} \cdot K_{EA,I}}) \cdot (E_t - AI_t) + 1]}{2 \cdot (\frac{1}{K_{E,A}} + \frac{1}{K_{E,I}} + \frac{1}{K_{E,A} \cdot K_{EA,I}})}$$

Assuming that E, AIE, EAI, and AEI have activity of 1, α_1, α_2, and α_3, respectively, the relative activity of the protease (A) can be described as:

$$A = \frac{E}{E_t} + \alpha_1 \cdot \frac{AIE}{E_t} + \alpha_2 \cdot \frac{EAI}{E_t} + \alpha_3 \cdot \frac{AEI}{E_t} = \frac{\frac{1}{AI} + \frac{\alpha_1}{K_{E,A}} + \frac{\alpha_2}{K_{E,I}} + \frac{\alpha_3}{K_{E,A} \cdot K_{EA,I}}}{\frac{1}{AI} + \frac{1}{K_{E,A}} + \frac{1}{K_{E,I}} + \frac{1}{K_{E,A} \cdot K_{EA,I}}}$$

And is a function of AI_t with a given E_t, $K_{E,A}$, $K_{E,I}$, $K_{EA,I}$, α_1, α_2, and α_3.
Inhibition max can be determined by 1-A when IJ approaches infinity:

$$1 - \frac{\frac{\alpha_1}{K_{E,A}} + \frac{\alpha_2}{K_{E,I}} + \frac{\alpha_3}{K_{E,A} \cdot K_{EA,I}}}{\frac{1}{K_{E,A}} + \frac{1}{K_{E,I}} + \frac{1}{K_{E,A} \cdot K_{EA,I}}}$$

And $\mathbf{IC_{50}}$ can be determined as IJ that resulted inhibition halfway towards inhibition max:

$$IC_{50} = \frac{1}{\frac{1}{K_{E,A}} + \frac{1}{K_{E,I}} + \frac{1}{K_{E,A} \cdot K_{EA,I}}}$$

In Figure 2a-c, we simulated enzyme inhibition curves with different $K_{EA,I}$ (ranging from 0.001 to 3), with the values of several constants obtained from Figure 1c:

1. $K_{E,I}$ = 50 nM, which is equivalent to the IC_{50} of free DAB (50nM) in the experimental setting.
2. $K_{E,A}$ = 1 nM, which is equivalent to the IC_{50} of free HD22 (1nM) in the experimental setting.
3. E_t=0.5 nM, which is the presumable thrombin concentration in the experimental setting.
4. α_1, α_2, and α_3 were determined to be 0.70, 0, and 0, respectively, which is determined by the relative activity of thrombin in the presence of saturating concentrations of HD22, DAB, and HD22-7A-DAB

In Figure 2e, $K_{EA,I}$ of different inhibitors were calculated by global fitting where α_1, α_2, and α_3 were set as constants to be 50nM, 1nM, 0.70, 0, and 0, respectively. $K_{E,I}$, $K_{E,A}$, Et were fitted as a shared parameter to be 0.71 nM, 45.0 nM, and 0.19 nM respectively. A good fitting with $K_{E,A}$ and $K_{E,I}$ similar to IC_{50} obtained from free aptamer and DAB binding implies that the linker does not significantly alter the affinity of either component. The fitted E_t is lower than the presumed thrombin concentration can be explained by partial loss of thrombin due to plate adsorption.
