# Supplementary material for: Aptameric hirudins as selective and reversible EXosite-ACTive site (EXACT) inhibitors
Source: Nat Commun. 2024 May 10;15:3977. doi: 10.1038/s41467-024-48211-6 (PMC11087511; doi:10.1038/s41467-024-48211-6)
Supplement: Supplementary file 3 — Reporting Summary [file 41467_2024_48211_MOESM3_ESM.pdf]

nature portfolio

Corresponding author(s): Sriram Krishnaswamy & Bruce A Sullenger

Last updated by author(s): Feb 29, 2024

Reporting Summary

Nature Portfolio wishes to improve the reproducibility of the work that we publish. This form provides structure for consistency and transparency in reporting. For further information on Nature Portfolio policies, see our Editorial Policies and the Editorial Policy Checklist.

Statistics

For all statistical analyses, confirm that the following items are present in the figure legend, table legend, main text, or Methods section.

n/a	Confirmed	
☐	☒	The exact sample size (*n*) for each experimental group/condition, given as a discrete number and unit of measurement
☐	☒	A statement on whether measurements were taken from distinct samples or whether the same sample was measured repeatedly
☐	☒	The statistical test(s) used AND whether they are one- or two-sided<br>*Only common tests should be described solely by name; describe more complex techniques in the Methods section.*
☒	☐	A description of all covariates tested
☐	☒	A description of any assumptions or corrections, such as tests of normality and adjustment for multiple comparisons
☐	☒	A full description of the statistical parameters including central tendency (e.g. means) or other basic estimates (e.g. regression coefficient) AND variation (e.g. standard deviation) or associated estimates of uncertainty (e.g. confidence intervals)
☐	☒	For null hypothesis testing, the test statistic (e.g. *F*, *t*, *r*) with confidence intervals, effect sizes, degrees of freedom and *P* value noted<br>*Give P values as exact values whenever suitable.*
☒	☐	For Bayesian analysis, information on the choice of priors and Markov chain Monte Carlo settings
☒	☐	For hierarchical and complex designs, identification of the appropriate level for tests and full reporting of outcomes
☒	☐	Estimates of effect sizes (e.g. Cohen's *d*, Pearson's *r*), indicating how they were calculated

Our web collection on statistics for biologists contains articles on many of the points above.

Software and code

Policy information about availability of computer code

Data collection	no software was used
Data analysis	Microsoft Excel 365, Graphpad Prism 9, OriginLab Pro, ImageLab 6.0.1, COOT, Phenix.Refine,PDBREDO

For manuscripts utilizing custom algorithms or software that are central to the research but not yet described in published literature, software must be made available to editors and reviewers. We strongly encourage code deposition in a community repository (e.g. GitHub). See the Nature Portfolio guidelines for submitting code & software for further information.

Data

Policy information about availability of data

All manuscripts must include a data availability statement. This statement should provide the following information, where applicable:

- Accession codes, unique identifiers, or web links for publicly available datasets
- A description of any restrictions on data availability
- For clinical datasets or third party data, please ensure that the statement adheres to our policy

Crystallographic data for the structures reported in this article have been deposited to the protein data bank with PDB ID 8TQS and will be available upon publication of the manuscript. The authors declare that other data supporting the findings of this study are available within the paper and its Supplementary Information files. Source data are provided. Should any raw data files be needed in another format they are available from the corresponding author upon reasonable request.

Research involving human participants, their data, or biological material

Policy information about studies with human participants or human data. See also policy information about sex, gender (identity/presentation), and sexual orientation and race, ethnicity and racism.

Reporting on sex and gender	We did not group our data by sex in this study, both male and female donors were recruited.
Reporting on race, ethnicity, or other socially relevant groupings	We did not use any socially relevant groupings in this study
Population characteristics	We did not consider any population characteristics in this study
Recruitment	Subjects were recruited from a pool of healthy adult volunteers within the Durham, NC area who responded to a posted advertisement. There was no limitation on sex, race, or other population characteristic. The only limitation is that the blood donor is not on medications that may affect clotting, such as NSAIDs. Self-selection/volunteer bias is possible as volunteers were given the option to choose to participate in the study in return for financial compensation. However, this self-selection is unlikely to impact the results as only healthy donors were studied and both sexes are included.
Ethics oversight	Duke University Health System (DUHS) Institutional Review Board for Clinical Investigations<br>Federalwide Assurance No: FWA 00009025<br>Protocol ID: Pro00007265

Note that full information on the approval of the study protocol must also be provided in the manuscript.

Field-specific reporting

Please select the one below that is the best fit for your research. If you are not sure, read the appropriate sections before making your selection.

[x] Life sciences [ ] Behavioural & social sciences [ ] Ecological, evolutionary & environmental sciences

For a reference copy of the document with all sections, see nature.com/documents/nr-reporting-summary-flat.pdf

Life sciences study design

All studies must disclose on these points even when the disclosure is negative.

Sample size	As all studies are biochemical, in vitro experiments, sample sizes were chosen to determine statistical significance based upon historical experience and determined empiraclly and based on our and others previous studies on the anticoagulant aptamers or small molecule inhibitors. Some references are provided: Nat. Biotechnol. 36, 606-613; Proc. Natl. Acad. Sci. U. S. A. 105, 5664-5669; Nature 419, 90-94 (2002);and J. Med. Chem. 45, 1757-1766 (2002).
Data exclusions	No data was excluded from analysis
Replication	All quantitative measurements were replicated 2 to 3 times as stated in the paper over two years and found to be reproducible
Randomization	For tests on purchased pooled plasma, no randomization is applicable as all assays were performed using the same plasma material. For tests on whole blood, no randomization was needed since we collected enough blood that all test conditions could be performed on each sample.
Blinding	As all data collection in this study was performed by instruments without subjective human-based measurements. No group allocation was need during data analysis as every sample was treated with all test conditions. Therefore, blinding was not performed.

Reporting for specific materials, systems and methods

We require information from authors about some types of materials, experimental systems and methods used in many studies. Here, indicate whether each material, system or method listed is relevant to your study. If you are not sure if a list item applies to your research, read the appropriate section before selecting a response.

Materials & experimental systems

n/a	Involved in the study
x	Antibodies
x	Eukaryotic cell lines
x	Palaeontology and archaeology
x	Animals and other organisms
x	Clinical data
x	Dual use research of concern
x	Plants

Methods

n/a	Involved in the study
x	ChIP-seq
x	Flow cytometry
x	MRI-based neuroimaging

Plants

Seed stocks	n/a
Novel plant genotypes	n/a
Authentication	n/a
